# *Haslea ostrearia* Pigment Marennine Affects Key Actors of Neuroinflammation and Decreases Cell Migration in Murine Neuroglial Cell Model

**DOI:** 10.3390/ijms24065388

**Published:** 2023-03-11

**Authors:** Sarah Méresse, Hélène Gateau, Tessa Tirnan, Vanessa Larrigaldie, Nathalie Casse, Pamela Pasetto, Jean-Luc Mouget, Stéphane Mortaud, Mostefa Fodil

**Affiliations:** 1 Immunologie et Neurogénétique Expérimentales et Moléculaires (INEM), UMR CNRS 7355, Université d’Orléans, 45071 Orléans, France; 2 Biologie des Organismes, Stress, Santé, Environnement (BiOSSE), Le Mans Université, Avenue Olivier Messiaen, 72085 Le Mans, France; 3 Institut des Molécules et Matériaux du Mans, UMR CNRS 6283, Le Mans Université, Avenue Olivier Messiaen, 72085 Le Mans, France

**Keywords:** diatoms, *Haslea ostrearia*, marennine, biomolecules, neuroglial cells, neuroinflammation, migration, immunostimulation

## Abstract

*Haslea ostrearia*, a cosmopolitan marine pennate diatom, produces a characteristic blue pigment called marennine that causes the greening of filter-feeding organisms, such as oysters. Previous studies evidenced various biological activities of purified marennine extract, such as antibacterial, antioxidant and antiproliferative effects. These effects could be beneficial to human health. However, the specific biological activity of marennine remains to be characterized, especially regarding primary cultures of mammals. In the present study, we aimed to determine in vitro the effects of a purified extract of marennine on neuroinflammatory and cell migratory processes. These effects were assessed at non-cytotoxic concentrations of 10 and 50μg/mL on primary cultures of neuroglial cells. Marennine strongly interacts with neuroinflammatory processes in the immunocompetent cells of the central nervous system, represented by astrocytes and microglial cells. An anti-migratory activity based on a neurospheres migration assay has also been observed. These results encourage further study of *Haslea* blue pigment effects, particularly the identification of molecular and cellular targets affected by marennine, and strengthen previous studies suggesting that marennine has bioactivities which could be beneficial for human health applications.

## 1. Introduction

Since a few decades, there has been an expanding interest in microalgae, notably due to their importance in aquatic ecosystems where they are responsible for most of the primary productivity. Indeed, microalgae form the basis of seafood webs and account for more than 50% of the oxygen produced yearly [1]. Those organisms involved not only in major ecosystem services but also in human health applications such as drug delivery, hypoxic tumor treatment and cancer therapy [2,3,4,5,6]. Due to their high metabolic flexibility, their ability to withstand various culture conditions, and their rapid growth, the number of studies on their use as a source of biologically valuable products has been rapidly increasing. For these reasons, the commercial production of bioactive compounds from microalgae is currently challenged by the biorefinery process. Currently, integrated microalgae cultivation technologies aimed at isolating biologically active substances from biomass, for cost-effective algae production, are being investigated. However, only a few strains of microalgae are actually exploited on an industrial scale [7].

On the one hand, microalgae are particularly valued as a source of antimicrobial, antiproliferative, anti-inflammatory, or antioxidant compounds [8,9,10,11]. On the other hand, microalgae can be a source of hazardous molecules for human health, such as toxins [12,13]. The beneficial or harmful effects of molecules derived from microalgae seem to depend on the strain cultivated and the environment in which the microalgae biomass is produced.

A large number of marine compounds have been shown to affect a wide variety of cellular and molecular targets, notably within the nervous systems [14,15]. For instance, in the central nervous system (CNS), carotenoid extracts from *Euglena gracilis* strongly dampered microglia activation and downregulated the mRNA expression levels and release of proinflammatory mediators, including interleukin-1 bêta (IL-1β), interleukin-6 (IL-6) and tumor-necrosis factor alpha (TNFα) in microglial cells [16]. In addition to that, an extract from the diatom *Phaeodactylum tricornutum* mainly composed of fucoxanthin has been shown to inhibit NF-κB and NLRP3 inflammasome activation induced by lipopolysaccharide (LPS) in bone marrow-derived immune cells as well as in astrocytes. Moreover, fucoxanthin decreases the production of major proinflammatory cytokines such as IL-1β, IL-6 and TNFα [17].

*Haslea ostrearia* is a cosmopolitan marine pennate diatom that can synthesize and release a water-soluble polysaccharidic blue-green pigment called marennine [18]. The name “marennine” was proposed by Lankester [19] in reference to Marennes-Oléron (Marennes, 45°49′25.8″ N 1°06′16.3″ W), a region of western France where oysters have been farmed for centuries. This pigment is responsible for the greening of oyster gills, a natural phenomenon of great importance for the oyster industry [20]. *Haslea ostrearia* is not only present on the French Brittany coasts but also within the Pacific Ocean on the Australian, American and Japanese coasts [21]. Until recently, green oysters were mainly consumed in France, but now they are also valued in these regions. Marennine is not involved in light capture or in photochemical activity [22], but it may confer photoprotective properties [23,24]. An ecological function can also be hypothesized due to allelopathic and antimicrobial effects exerted by marennine, thus giving *Haslea* cells a competitive advantage over other microalgae or bacteria [21,25].

At present, the complete bioactive potential of this pigment is still largely unknown. It has already been described that aqueous extracts containing marennine exhibited anticoagulant properties using human plasma [26]. This study also reported that marennine displayed in vitro growth-inhibiting properties of Herpes Simplex Virus type 1 (HSV-1) in Vero cells [26]. Gastineau et al. [27] confirmed the antiviral effects of purified marennine against the HSV-1 herpes virus with an EC50 of 27 µg/mL, which was twice as effective as the reference drug Zovirax. Moreover, marennine has demonstrated anti-oxidative effects superior to strong anti-oxidative molecules, such as ascorbic acid and Trolox, usually used in the food industry [24]. In the context of anti-cancer therapies, different marennine forms varying in the purification methodology have demonstrated an anti-proliferative effect on human cancer cell lines, such as SKOV-3 (ovarian cancer), SW116 (colon cancer) or M113 (melanoma) [27,28]. These pioneer studies highlighted the potential of marennine as a biologically active organic compound for carcinotherapy, but they were carried out only on tumor cell models.

The present work aimed to investigate the effects of marennine on primary culture cellular models, focusing particularly on the immunocompetent cells of the CNS. To this end, the impact of marennine exposure at 10, 50 and 100 μg/mL on murine primary culture of astrocytes and microglial cells was investigated to check for possible effects on cell viability. In order to determine if marennine acts on neuroinflammatory processes, its effect was investigated at the level of gene expression, focusing on the main inflammatory cytokines. The inflammatory status of populations of immunocompetent cells, astrocytes and microglial cells was also determined following exposure to marennine. Lastly, the effect of marennine was assessed on proinflammatory cytokine release IL-1β, IL-6, TNFα and on the anti-inflammatory cytokine interleukine-10 (IL-10).

## 2. Results

### 2.1. Marennine Is Not Cytotoxic at 10 µg/mL and 50 µg/mL

**Figure 1:**
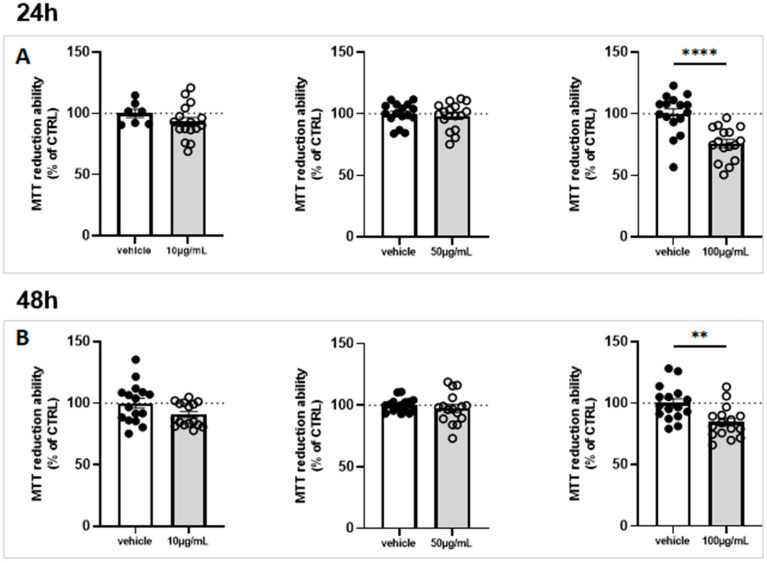
Effects of 10, 50 and 100 µg/mL of marennine concentration on cell viability in neuroglial cell culture. Cell viability was determined using MTT assay. (**A**) After 24 h and (**B**) after 48 h of marennine exposure. For a 24 h exposure, 10 µg/mL vehicle n = 7, marennine = 16; 50 µg/mL vehicle n = 16, marennine n = 16; 100 µg/mL vehicle n = 16, marennine n = 16. For a 48 h exposure, 10 µg/mL vehicle n = 16, marennine n = 16; 50 µg/mL vehicle n = 16, marennine n = 16; 100 µg/mL vehicle n = 16, marennine n = 16. Values represent mean ± SEM. Statistical differences from control are indicated as follows: ** *p* < 0.01; **** *p* < 0.0001 using Mann–Whitney test.

The effect on cell viability of marennine at 10, 50 and 100 µg/mL was determined in co-culture of astrocytes and microglial cells. This was evaluated after 24 h and 48 h of exposure. Marennine did not affect the cell viability at 10 and 50 µg/mL (Figure 1A,B). At 100 µg/mL, however, results showed that marennine decreased cell viability by ca. 75% after 24 h (Figure 1A) and 85% after 48 h (Figure 1B). To prevent cytotoxic effects in further experiments, the concentration of 100 µg/mL was excluded.

### 2.2. Marennine Induces an Upregulation of Immunomodulatory Genes

**Figure 2:**
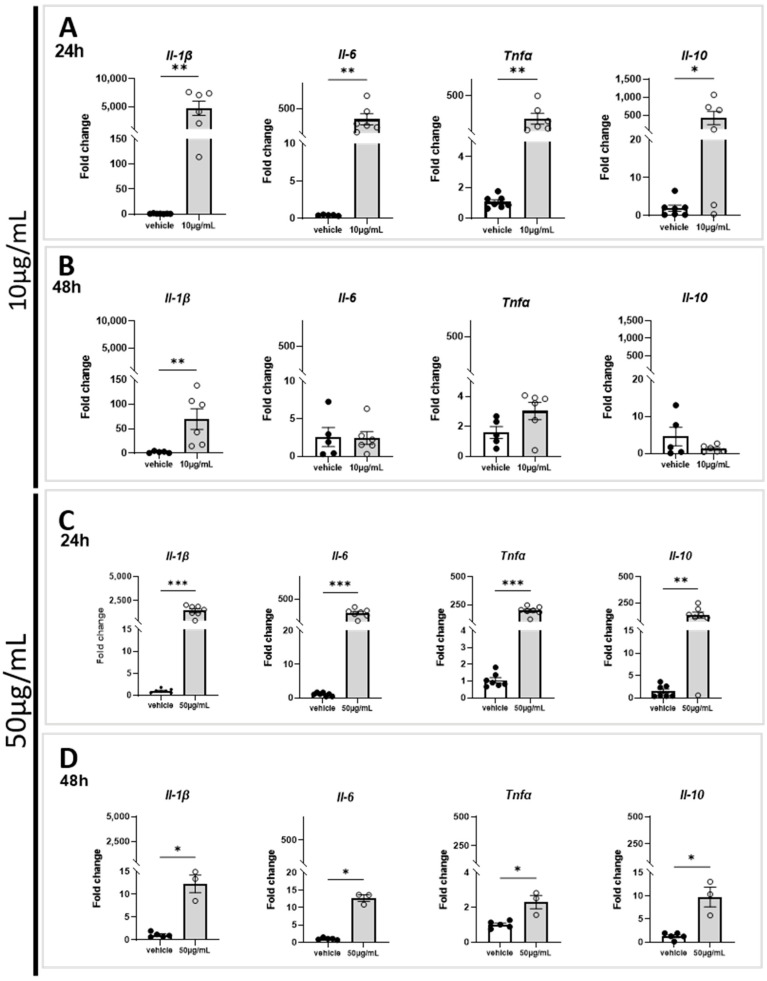
Effects of 10 and 50 µg/mL of marennine on gene expression in astrocytes and microglial cells after 24 h and 48 h of treatment. Levels of gene expression *Il-1β, Il-6*, *Tnfα* and *Il-10* after marennine exposure were measured by RT-qPCR. (**A**) After 24 h of marennine with 10 µg/mL. (**B**) After 48 h of marennine with 10 µg/mL. (**C**) After 24 h of marennine with 50 µg/mL. (**D**) After 48 h of marennine with 50 µg/mL. At 10 µg/mL, for 24 h *Il-1β*: vehicle n = 5, marennine n = 6; *Il-6*: vehicle n = 7, marennine n = 6; *Tnfα*: vehicle n = 7, marennine n = 6; *Il-10*: vehicle n = 7, marennine n = 6. For 48 h *Il-1β*: vehicle n = 5, marennine n = 6; *Il-6*: vehicle n = 5, marennine n = 6; *Tnfα*: vehicle n = 5, marennine n = 6; *Il-10*: vehicle n = 5, marennine n = 6. 50 µg/mL, for 24 h *Il-1β*: vehicle n = 7, marennine n = 7; *Il-6*: vehicle n = 7, marennine n = 7; *Tnfα*: vehicle n = 7, marennine n = 7; *Il-10*: vehicle n = 7, marennine n = 7. For 48 h *Il-1β*: vehicle n = 5, marennine n = 3; vehicle n = 5, marennine n = 3; *Tnfα*: vehicle n = 5, marennine n = 3; *Il-10*: vehicle n = 5, marennine n = 3. Values represent mean ± SEM. Statistical differences from the control are indicated as follows: * *p* < 0.05; ** *p* < 0.01; *** *p* < 0.001 using Mann–Whitney test.

The effect of marennine at 10 and 50 µg/mL concentrations was tested on gene expression involved in the neuroinflammatory process. At 10 µg/mL, marennine induced a strong upregulation of proinflammatory genes *Il-1β*, *Il-6*, *Tnfα* and of the anti-inflammatory gene *Il-10* after 24 h of exposure (Figure 2A), and an upregulation only for *Il-1β* gene expression (Figure 2B) after 48 h. It is worth noting that the upregulation of both proinflammatory and anti-inflammatory genes after 24 h of treatment was more significant than after 48 h. At 50 µg/mL, marennine exposure resulted in a strong upregulation of the proinflammatory genes *Il-1β*, *Il-6*, *Tnfα* and of the anti-inflammatory gene *Il-10* (Figure 2C). The effects were less significant than at 10 µg/mL.

After 48 h of treatment, the modulation of gene expression was lower than after 24 h of marennine exposure, but *Il-1β*, *Il-6*, *Tnfα* and *Il-10* remained upregulated (Figure 2D). The upregulation of inflammatory genes depends on the concentration and duration of treatment.

### 2.3. Marennine Affects the Population of Immunocompetent Cells of the CNS

As astrocytes and microglial cells are considered the main actors of the neuroinflammatory process, the effect of marennine on these cell models was assessed after 24 h and 48 h of treatment at 10 and 50 µg/mL.

#### 2.3.1. Marennine Modifies the Inflammatory Status without Affecting the Total Amount of Astrocyte Cells

**Figure 3:**
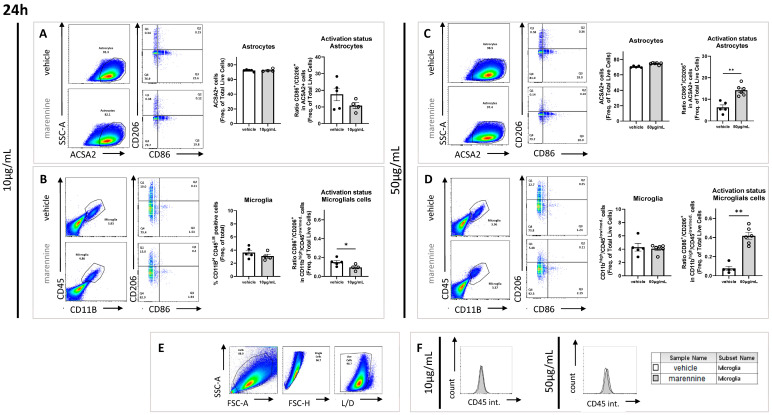
Effects of 10 and 50 µg/mL of marennine on astrocytes and microglial cells after 24 h of exposure. (**A**) Astrocytes were gated as ACSA2 positive; activation status was determined through CD86 and CD206 staining after exposure to 10 µg/mL marennine. (**B**) Microglial cells were gated as CD11b^high^ and CD45^low/medium;^ activation status was determined through CD86 and CD206 staining after exposure to 10 µg/mL marennine. (**C**) Astrocytes were gated as ACSA2 positive; activation status was determined through CD86 and CD206 staining after marennine exposure at 50 µg/mL. (**D**) Microglial cells were gated as CD11b^high^ and CD45^low/medium;^ activation status was determined through CD86 and CD206 staining after marennine at 50 µg/mL. (**E**) Cell populations and activation status were determined by flow cytometry. A gate was first created on non-debris populations, excluding very low SSC/FSC events; cells are then gated on single cells and selected by Live/Dead staining. (**F**) Quantification of CD45 intensity after a marennine of 10 µg/mL (left) and 50 µg/mL (right). For 24 h, at 10 µg/mL vehicle n = 5, marennine n = 4. At 50 µg/mL vehicle n = 5, marennine n = 6. Values represent mean ± SEM. Significant differences from control are indicated as follows: * *p* < 0.05; ** *p* < 0.01 using Mann-Whitney test.

**Figure 4:**
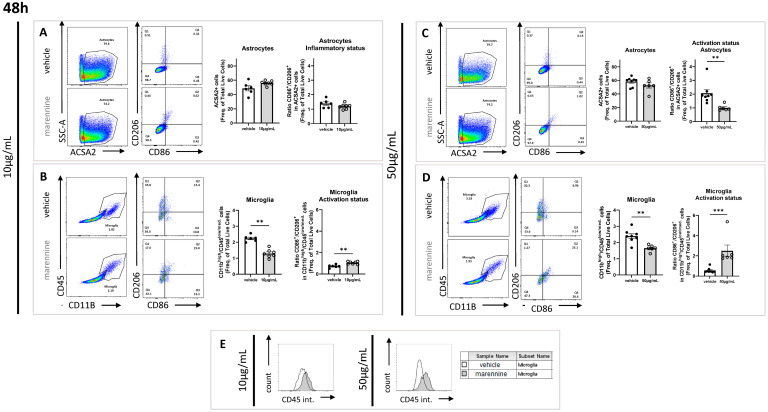
Effects of 10 and 50 µg/mL of marennine in astrocytes and microglial cells after 48 h of exposure. (**A**) Astrocytes were gated as ACSA2 positive; activation status was determined through CD86 and CD206 staining after exposure to marennine at 10 µg/mL. (**B**) Microglial cells were gated as CD11b^high^ and CD45^low/medium;^ activation status was determined through CD86 and CD206 staining after exposure to marennine at 10 µg/mL. (**C**) Astrocytes were gated as ACSA2 positive; activation status was determined through CD86 and CD206 staining after exposure to marennine at 50 µg/mL. (**D**) Microglial cells were gated as CD11b^high^ and CD45^low/medium;^ activation status was determined through CD86 and CD206 staining after exposure to marennine at 50 µg/mL. (**E**) Quantification of CD45 intensity after a marennine of 10 µg/mL (left) and 50 µg/mL (right). For 24 h, at 10 µg/mL vehicle n = 6, marennine n = 7. At 50 µg/mL vehicle n = 8, marennine n = 6. Values represent mean ± SEM. Statistically differences from control are indicated as follows: ** *p* < 0.01: *** *p* < 0.001 using Mann-Whitney test.

ACSA2 (astrocyte cell surface antigen-2) staining was used to label astrocyte cell types. To investigate the activation status of those immunocompetent cells, CD86 was used as a costimulatory marker of proinflammatory status, and the mannose receptor CD206 was used as a marker of the anti-inflammatory status. At 10 µg/mL, following 24 h and 48 h of exposure, marennine did not affect the percentage of astrocyte populations represented by the ACSA2 positive cells on the total amount of cells, nor did it affect their activation status, as illustrated by the ratio CD86^+^/CD206^+^ (Figure 3A and Figure 4A). At 50 µg/mL, following 24 h of exposure, marennine still did not affect the percentage of astrocyte populations. Nevertheless, a proinflammatory status in the astrocyte populations was observed (Figure 3C). After 48 h of treatment, the population was not impacted, and the anti-inflammatory process took place (Figure 4C).

#### 2.3.2. Marennine Modifies the Inflammatory Status and Modulates the Total Amount of Microglial Cells

CD11b and CD45 staining was used to study microglial cell responses. As for astrocytes, their activation status was investigated using CD86 as a marker of proinflammatory status and CD206 as a marker of anti-inflammatory cells. After 24 h exposure to 10 µg/mL marennine, no effect was observed on the microglial cell population percentage (Figure 3B), however microglial cells showed an anti-inflammatory response (Figure 3B). Following 48 h of exposure, marennine decreased the populations of microglial cells and the macrophages displayed proinflammatory status (Figure 4B). The effect of marennine on neuroinflammation seems to depend on the exposure duration. At 50 µg/mL and after 24 h of exposure, the percentage of microglial cell populations was not affected by marennine, and the activation status seems more likely to be proinflammatory (Figure 3D). After 48 h of exposure to marennine, microglial cells remained in proinflammatory status, with a decrease in the population (Figure 4D). Moreover, the intensity of the staining of CD45 in the population of microglial cells exposed to marennine was higher (Figure 3B,D and Figure 4B,D) and its shifts means that the phenomenon is time and concentration dependent (Figure 3F and Figure 4E).

### 2.4. Marennine Induces a Release of Key Actors of Neuroinflammation

**Figure 5:**
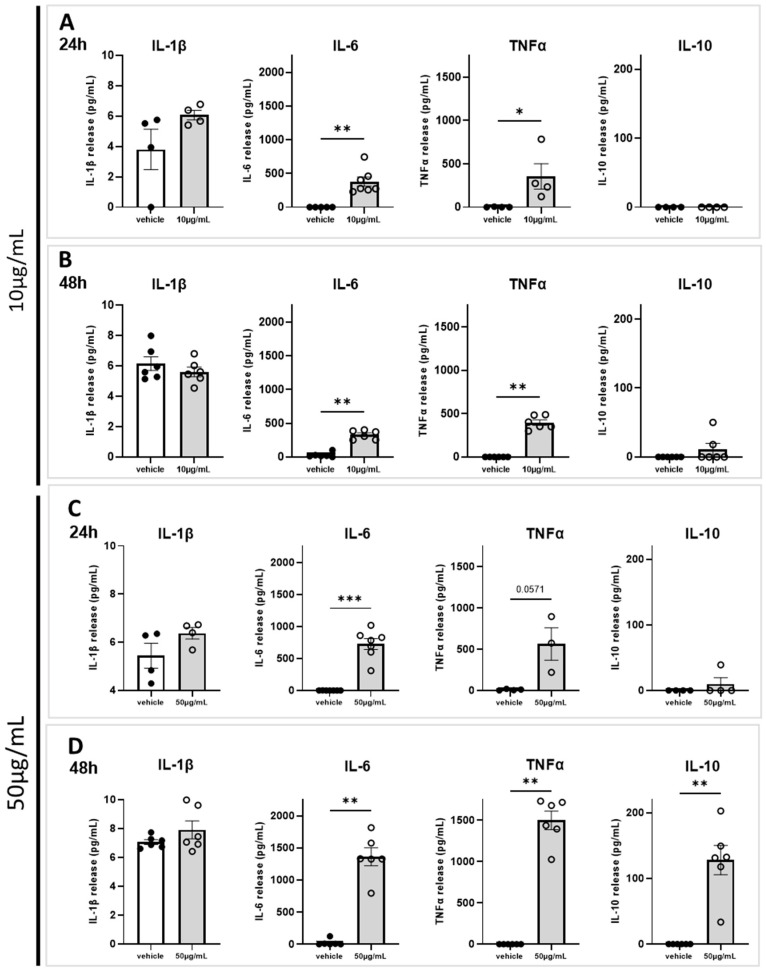
Effects of 10 and 50 µg/mL of marennine on cytokine release in astrocytes and microglial cells after 24 h and 48 h of treatment. Levels of cytokines IL-1β, IL-6, TNFα and IL-10 release in the supernatant after treatment were measured by ELISA-assay. (**A**) After 24 h of treatment with 10 µg/mL. (**B**) After 48 h of treatment with 10 µg/mL. (**C**) After 24 h of treatment with 50 µg/mL. (**D**) After 48 h of treatment with 50 µg/mL. At 10 µg/mL, for 24 h IL-1β: vehicle n = 4, marennine n = 4; IL-6: vehicle n = 5, marennine n = 7; TNFα: vehicle n = 4, marennine n = 4; IL-10: vehicle n = 4, marennine n = 4. For 48 h IL-1β: vehicle n = 6, marennine n = 6; IL-6: vehicle n = 6, marennine n = 6; TNFα: vehicle n = 6, marennine n = 6; IL-10: vehicle n = 6, marennine n = 6. At 50 µg/mL, for 24 h IL-1β: vehicle n = 4, marennine n = 4; IL-6: vehicle n = 7, marennine n = 7; TNFα: vehicle n = 4, marennine n = 3; IL-10: vehicle n = 4, marennine n = 4. For 48 h IL-1β: vehicle n = 6, marennine n = 6; IL-6: vehicle n = 6, marennine n = 6; TNFα: vehicle n = 6, marennine n = 6; IL-10: vehicle n = 6, marennine n = 6. Values represent mean ± SEM. Significant differences from control are indicated: * *p* < 0.05; ** *p* < 0.01; *** *p* < 0.001 using Mann–Whitney test.

This series of experiments was aimed to determine the effect of marennine at concentrations of 10 and 50 µg/mL on the release of inflammatory cytokines, in particular the key proinflammatory cytokines IL-1β, IL-6 and TNFα. Furthermore, the release of IL-10 was to evaluated to assess the anti-inflammatory response. No significant modification in IL-1β release was observed at any marennine concentration or exposure duration (Figure 5A–D). In contrast, a significant release of IL-6 and TNFα was observed in the supernatant, which increased with marennine concentration and exposure duration, whereas they were undetectable in the vehicle condition (Figure 5A–D). The concentration of IL-10 was hardly detectable in the supernatants (Figure 5A–C), except for a massive release observed at the highest concentration and the longest exposure (Figure 5D). Thus, the release of both proinflammatory and anti-inflammatory cytokines is dependent on marennine concentration and duration of exposure, with the highest released observed at 50 µg/mL and 48 h of treatment.

### 2.5. Marennine Reduces the Migration Surface of Cells from Neurospheres without Inducing Cytotoxicity

**Figure 6:**
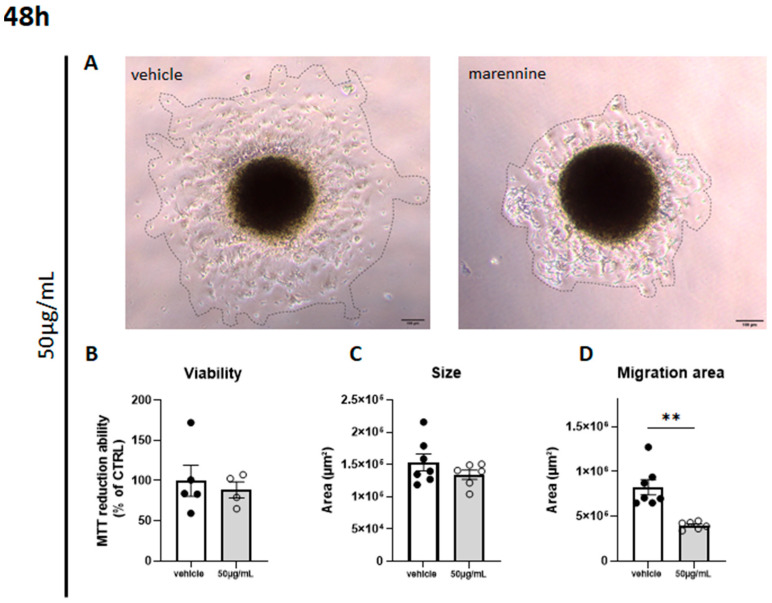
Effects of 50 µg/mL marennine on cell migration after 48 h exposure. (**A**) Representative images were taken using a 10× objective of a phase-contrast microscope; the outer dark circles in the phase-contrast images indicate the area covered by the migrated cells. (**B**) Cell viability was determined using MTT assay; vehicle n = 5, marennine n = 4. (**C**) Quantification of neurosphere size after migration; vehicle n = 7, marennine n = 6. (**D**) Quantification of the migration area covered by migrating cells from the core of the neurosphere; vehicle n = 7, marennine n = 6. Scale bar = 100 µm. Values represent mean ± SEM. Significant differences from control are indicated: ** *p* < 0.01.

Three-dimensional culture models are increasingly used in research for the study of the nervous system. These models are closer to reality and demonstrate less sensitivity to treatment due to their organization [29]. Moreover, cerebral organoids allow for higher differentiation of NSCs, notably into differentiated astrocytes. The neurospheres migration assay is a useful in vitro bio-tool that reveals the neural cell migration process [30]. In the present work, the neurosphere migration assay demonstrated that exposure to 50 µg/mL of marennine did not affect either the viability or the size of cerebral organoids (Figure 6A,B). Furthermore, a significant reduction of the cell migration area (around 50%) was observed for the 48 h of treatment (Figure 6D).

## 3. Discussion

Astrocytes are the most abundant glial cells in the CNS, maintaining homeostasis and supporting the function of neurons through trophic and metabolic support [31]. Meanwhile, microglial cells are considered as the resident macrophages of the brain [32]. Both cell types strongly interact with each other and are considered as the immunocompetent cells regarding their capacity to mediate immune responses.

In Th1-type immune responses, the key proinflammatory cytokines are represented by IL-1β, IL-6 and TNFα, which play a critical role in modulating the immune system. Th2-type immune responses, known to be anti-inflammatory, are notably characterized by the production of IL-10 cytokines [33,34]. TNFα, initially discovered as a result of its antitumor activity, has been shown to be one of the major mediators of inflammation [35]. However, it has been demonstrated in a TNFR-deficient mice model that TNFα acts to damper the inflammatory response and plays a role in the repair process of inflammation [36,37]. While TNFα can be produced by both astrocytes and microglial cells, IL-6 and IL-1β are mainly released by astrocytes and microglial cells, respectively. Since crosstalks between microglial cells and astrocytes are crucial for the behaviors of both cell types, a primary co-culture of those inflammatory cells is more representative of physiological condition than immortalized monoculture cell lines [38].

In the present study, the quantification of cytokines release illustrated the time- and concentration-dependent effects of marennine on neuroinflammatory processes. In particular, co-cultures of astrocytes and microglial cells demonstrated that marennine at non-cytotoxic concentrations induces an upregulation of both proinflammatory (*Il-1β*, *Il-6*, *Tnfα*) and anti-inflammatory (*Il-10*) genes. The inflammatory response at the gene expression level was higher at 10 µg/mL than at 50 µg/mL after 24 h of exposure. After 48 h of treatment, a significant upregulation of *Il-1β* at 10 µg/mL can be noticed, and at 50 µg/mL, the response induced by marennine also involved *Il-6*, *Tnfα* and *Il-10*. These results suggest that the expression of *Il-1β* is differentially regulated compared to other inflammatory genes after marennine exposure. Moreover, despite strong upregulation of *Il-10* gene expression, this cytokine can only be detected in the supernatant at the highest concentration and longest exposure to marennine. Hence, we showed for the first time that *Haslea* blue pigment activates the inflammatory pathway and interacts with glial cells of the CNS. Given the importance of marennine for the aquaculture industry, the temporal kinetics of both pro- and anti-inflammatory signaling after marennine exposure will be studied further. For instance, the heterogeneous kinetics of the immune response following a neuroinflammatory injury has already been described in vitro using the RAW 264.7 macrophages cell line [39]. These authors also reported that the proinflammatory-like response began as soon as 1 h after LPS exposure, whereas anti-inflammatory responses, represented by IL-10, started 10 h after LPS-induces inflammation [39]. As a reminder, LPS is a bacterial endotoxin that specifically promotes the production and release of inflammatory cytokines [40].

It has to be noted that despite upregulation of the *Il-1β* gene following 24 h and 48 h of treatment at 10 and 50 µg/mL, no significant difference in cytokine release can be observed. This can be due to notable IL-10 upregulation leading, as an anti-inflammatory cytokine, to the inhibition of IL-1β synthesis [41,42]. This can also be explained by smaller proportions of microglial cells in culture, when compared to basal release in the control condition. Indeed, in the flow cytometry experiments, microglial cells represented less than 4% of the total population of neuroglial cells, which is close to in vivo conditions in the mammalian brain [43]. Yet, we have to take into account that for the release of IL-1β into extracellular space, pro-IL-1β needs to be cleaved by the protease caspase-1 [44]. The activation of caspase-1 occurs via recruitment to a multi-protein complex termed the inflammasome, especially involving the NLRP3 protein. To further study the difference in IL-1β response, the effective synthesis of pro-IL-1β, then the mechanisms of its release, should be investigated at the molecular level.

If conflicting reports exist about the capacity of microglial cells to produce IL-10, astrocytes are known to respond to IL-10 and then attenuate microglial activation [45,46,47]. We thus aimed at determining the effects of marennine on immunocompetent cell populations and activation. The activation status of these immunocompetent cells is referred to as the balance ratio between CD86^+^ and CD206^+^ [48,49]. Whatever marennine concentration, no harmful effect could be observed after 24 h of exposure, neither on astrocytes nor microglial cell population, at 10 or 50 µg/mL. Nevertheless, a non-canonical effect of marennine on the inflammatory status can be hypothesized. On the one hand, a balance more likely toward an anti-inflammatory status at 10 µg/mL is observed in the microglial population; on the other hand, the balance ratio at 50 µg/mL indicates an activation facing proinflammatory processes. After 48 h of exposure, this population decreased at all concentrations, and a proinflammatory status is developed. Concerning astrocytes, the main glial cells responsible for the homeostasis of the CNS, the percentage of the population is not affected at both concentrations, whatever the exposure duration. Based on the balance ratio between CD86^+^ and CD206^+^, at 50 µg/mL marennine exposure, a proinflammatory status is first observed during the first 24 h, shifting to an anti-inflammatory status after 48 h. These results underline that following an in vitro treatment with marennine, the two main types of immunocompetent CNS cells do not have the same kinetics and the same inflammatory responses. This can be supported by the fact that activated microglial cells and reactive astrocytes may achieve immune “stimulation” through mutual communication and cooperation in neuroinflammation [45].

Activated and resting microglia can be separated based on relative levels of CD45 expression. Moreover, in vivo studies have demonstrated that minocycline, an anti-inflammatory treatment of the CNS, significantly reduced the percentage of CD45^high^ microglia [50]. Nevertheless, in vitro studies using human or rodent cells suggest that CD45 can downregulate microglial activation [51]. For example, murine microglia devoid of CD45 expression demonstrate an over-activated phenotype [52,53]. CD45 also downregulates HIV-1 replication in microglia, suggesting a neuroprotective effect [51]. CD45 staining appears to be a biomarker of proinflammatory status leading to the establishment of neuroprotective inflammatory response. In this study, we demonstrate an increase in the intensity of CD45 staining in a concentration- and time-dependent manner. However, we have to keep in mind the establishment of an anti-inflammatory response, after 48 h of exposure, mediated by IL-10.

Proliferating progenitor cells and glial cells tend to migrate from the neurogenic niches toward their final destination in the brain. Previous studies have shown that inflammation can disturb neuronal and non-neuronal cells migration and thus disrupt neurodevelopment [54,55]. This alteration can also favor the onset of neurodegenerative diseases later in life [56]. Our results demonstrate that a purified extract of marennine strongly inhibits cell migration in a 3D neurosphere model. It is known that inhibition of cell migration from neurogenic niches in response to environmental compounds exposure has been correlated with cytoskeletal disruption [57]. This could be of great importance, as few anti-cancer drugs are known to target the cytoskeleton to fight against metastatic dissemination such as snake toxins [58] and vincristine, an alkaloid extracted from Madagascar periwinkle [59]. Moreover, while a cascade of cytokines can be beneficial for the host by initiating the inflammatory response, a dysregulation of the balance between pro-inflammatory and anti-inflammatory mediators can be deleterious. Here, we highlight an immunostimulatory effect of marennine, knowing that the pro-inflammatory status can be linked to neurotoxic effects, while the anti-inflammatory status is generally associated with neuroprotective effects [56].

## 4. Materials and Methods

### 4.1. Animals

C57BL/6JRj breeding mice (Janvier Labs, Le Genest-St-Isle, France) were housed in a temperature- and humidity-controlled environment on a 12 h light/dark cycle with ad libitum access to food and water.

### 4.2. Cultivation of Microalgae and Marennine Extraction

*Haslea ostrearia* diatoms used for biomass production and pigment extraction were derived from samples collected in Bourgneuf Bay, France (46°59′19′′ N/2°14′14′′ W). The blue pigment was extracted and purified as previously described [60]. To sum up, *Haslea ostrearia* strain Nantes Cultures Collection (NCC) 495 was cultured at 16 ± 1 °C, with an irradiance of 100 µmol·m^−2^·s^−1^ provided by Philips TLD 36 W/965 fluorescent tubes with an alternance cycle of 14 h light/10 h dark. Cultures were grown with autoclaved artificial seawater, prepared from a commercial sea salt mix (Instant Ocean, Aquarium Systems^®,^ Mentor, OH, USA), pH 7.6 ± 0.2, salinity 32 ppm, with an enrichment solution as described by Mouget et al. [61].

The culture medium was then filtered through 15 μm and 1.4 μm paper filters (Grosseron, Coueron, France) to remove residues. A specific precipitation method was followed, using acid and base according to the procedure described in the patent n° (FR2019/052933), to concentrate the filtered supernatant. The blue precipitate formed was gathered by centrifugation (4000 rpm, for 5 min) and dissolved with formic acid. The blue concentrated extract was dialyzed, and the deep blue retentate was further purified. The marennine, which interacted with the C-18 cartridge, was recovered with a water-ethanol 1:1 mixture. The mixture was then evaporated in order to recover the purified extract of marennine as a dry powder, which was then solubilized with sterile water at a stock concentration of 1 mg/mL. Purified pigment was stored, protected from light, at 4 °C.

### 4.3. Neuroglial Cell Culture and Marennine Exposure

In vitro murine primary cultures of astrocytes and microglial cells were performed from postnatal days 1 to days 3 C57BL/6JRj mice. Neuroglial cells were cultured according to the modified experimental procedures of Schildge and colleagues [62]. Four to eight male and female brains were used to perform each cell culture. Briefly, to obtain a single-cell suspension, the olfactory bulbs and the cerebellum were removed, and the meninges were peeled off. The dissected telencephalon was mechanically dissociated. The cells were then resuspended in DMEM medium D5671 (Sigma, Saint-Louis, MO, USA), completed with 10% FBS (GE Healthcare HyClone, Logan, UT, USA), 1000 units/mL Penicillin-Streptomycin 15070-063(Gibco, Waltham, MA, USA) and 2 mM of L-Glutamine 25030-024 (GIBCO, Waltham, MA, USA). Cells were enumerated and plated on appropriate plates, and then maintained in cultures at 37 °C with 5% CO_2_ in a humidified incubator. The first change of medium was after four days of culture, and then each two days. Cells were treated with marennine at 10, 50 and 100 µg/mL during 24 h and 48 h.

### 4.4. Neuroglial Cell Viability Analysis

Cell viability was measured by the 3-(4,5-dimethyl-2-yl)22,5-diphenyl-2H-tetrazolium bromide M2003 (MTT, Sigma, Saint-Louis, MO, USA) assay. Viability was measured after 24 h and 48 h of treatment in the primary co-culture of astrocytes and microglial cells. Briefly, cells were plated at 4 × 10^4^ cells per well in 96-well plates. At the end of exposure, MTT was added to the medium at a concentration of 5 mg/mL and incubated at 37 °C for three hours. The formazan product generated was solubilized in dimethyl sulfoxide for 15 min and measured at 570 nm using a microplate reader (ClarioStar, BMG LABTECH, Cary, NC, USA).

### 4.5. Gene Expression Analysis

Quantitative PCR was used to quantify gene expression. Gene mRNA expressions were performed using GoTaq^®^qPCR Master Mix A5002 (Promega, Madison, WI, USA). Reverse transcription of RNA into cDNA was carried out with GoScript Reverse Kit A5001 (Promega, Madison, WI, USA). RT-qPCR was performed with Fast SYBR Green master mix on an ARIA MX (Stratagene MX3005P, Agilent technologies, Santa Clara, CA, USA). All primer sequences used were from Qiagen (Valencia, CA, USA): *Il-6* (#QT00098875), *Il-10* (#QT00106169), *Tnfα* (#QT00104006) and *Il-1β* (#QT01048355). RNA expression was normalized to *18S* expression (#QT02448075) and *β-actin* (#QTQT00095242). Data were analyzed using the comparative analysis of relative expression by ΔΔCt methods. For all experiments, biological triplicate and technical triplicate were performed.

### 4.6. Neuroglial Cell Populations and Activation Status Analysis

Firstly, flow cytometry was used to determine the effect of the blue pigment marennine on the population of the immuno-competent cells of the CNS, astrocytes and microglial cells. Second, we determined the activation status of those cells. Cells were plated at 1 × 10^5^ cells per well in 6-well plates. After 24 h and 48 h of exposure, cells were collected and stained with extracellular conjugated antibodies: Fixable Viability Dye 65-0865-14, 1/800 (eBiosciences™, San Diego, CA, USA), anti-ACSA2 APC 130-117-386, 1:50 (MILTENYI Biotec, Bergisch Gladbach, ), anti-CD45 V450 560501, 1:100 (BD Horizon™, San Jose, CA, USA,), anti-CD11b PerCP/Cy5 560993, 1:100 (BD Pharmingen™, San Jose, CA, USA), anti-CD86 FITC 561961, 1:100 (BD Biosciences, Heidelberg, Germany) and anti-CD206 PE-Cy7 141720, 1:100 (BioLegend, San Diego, CA, USA™). Non-conjugated anti-CD16/32 antibody 553142, 1:100 (BD Pharmingen™, San Jose, CA, USA) was used to block non-specific Fc receptors for 20 min at 4 °C. Then, cells were washed and resuspended in a lysis solution (BD FACS™ Lysing Buffer, BD Biosciences, Heidelberg, Germany) and maintained at 4 °C before acquisition the following day. Data were collected with a flow cytometer (BD FACSCanto II, BD Biosciences, Heidelberg, Germany) and analyzed with FlowJo v10 software^®^ (Tree Star, Ashland, OR, USA). Very low SSC and very low FSC were considered as cellular debris and excluded to define the populations of interest. ACSA2^+^ cells were considered as astrocytes. CD11b^high-medium^/CD45^low^ cells were gated as microglial cells. Activation status was determined using CD86 as a costimulatory marker of proinflammatory status and man-nose receptor CD206 as a marker of anti-inflammatory status [63]. To illustrate the balance between proinflammatory and anti-inflammatory status, we represented the balance ratio of CD86^+^/CD206^+^. For all experiments, at least three biological replicates were performed.

### 4.7. Measurement of Cytokine Release

For cytokine release determination, supernatants from astrocytes and microglial cell cultures were taken after 24 h and 48 h of exposure. Extracellular ATP was added at 100 µM during 30 min to stimulate IL-1β secretion. Samples were stored at −20 °C until analyzed. Release of cytokines was analyzed by ELISA assay kits for murine IL-1β, IL-6, TNFα and IL-10 (R&D system, Minneapolis, MN, USA) according to the manufacturer’s instructions. For all experiments, at least three biological replicates and three technical replicates were performed.

### 4.8. Neurospheres Migration Assay

Neural stem cells (NSCs) composing the neurospheres were cultured using adapted protocols [64,65]. Cell viability was assessed using the MTT assay from adapted protocols of Bresciani et al. [66]. NSCs were first proliferated in standard DMEM medium supplemented with epidermal growth factors E9644 (EGF, Sigma, Saint-Louis, MO, USA) until confluence was reached (about 5–6 days). Cells were detached using Trypsin-EDTA 25200-056 (Gibco, Waltham, MA, USA) and seeded in 96-well Corning Ultra low attachment plates 7007 (Corning Inc., Corning, NY, USA) at 1.25 × 10^6^ cells. The medium was changed every two days by removing half of the medium, and the plates were shaken at 150 rpm for 10 min before adding fresh DMEM medium enriched with EGF. Once neurospheres were well-formed, the medium was replaced with DMEM/F12 21331-020 (Gibco, Waltham, MA, USA) containing 1% P/S supplemented with 1% N2 17502-048 (Gibco, Waltham, MA, USA) to allow differentiation into neuroglial cells. Neurospheres were then transferred into a 24-well plate with a glass coverslip coated with Poly-L-Lysine P7405 (PLL, Sigma, Saint-Louis, MO, USA) and matrigel^®^ 356237 (BD Biosciences, Heidelberg, Germany) to allow migration.

Attached neurospheres were non-exposed (vehicle condition) or treated with 50 µg/mL of a purified extract of marennine from *Haslea ostrearia* during 48 h. These conditions correspond to the highest values of concentration and exposure duration used on 2D cultures. Phase-contrast photographs of each well were taken by a microscope using a 10× objective (Nikon ECLIPSE TS2, Nikon Inc., Melville, NY, USA). Measurements were performed according to the protocol previously described [67]. Briefly, to assess the size of each neurosphere, the area was measured. To determine the migration of cells, we measured the area covered by migrated cells using the Image J^®^ software (NIH Image, Bethesda, MD, USA [68]).

### 4.9. Statistics

The results are presented as mean ± standard error of the mean (SEM) for each experimental group. Each point corresponds to biological replicates. Assuming that our data did not follow the normal law of distribution, differences compared with the control group were analyzed by Mann–Whitney tests for non-parametric data using GraphPad Prism 9^®^ (GraphPad Software, San Diego, CA, USA). *p*-values ≤ 0.05 were considered statistically significant.

## 5. Conclusions

Marennine has been highlighted in previous studies as a potential biologically active compound that can be benefit in pathological context [23,24,27,69]. In the present study, we demonstrated, for the first time, that marennine has a time- and concentration-dependent significant effect on dynamic changes of neuroinflammatory processes. These findings suggest an interesting role of marennine in immunomodulation by regulating intercellular communications between astrocytes and microglial cells. Nevertheless, in vivo experiments are needed to study the temporal kinetics of both pro- and anti-inflammatory signaling after marennine exposure and determine its physiological effects on the mammalian CNS. Marennine also exhibits an anti-migratory property. This could be of great interest when studying marennine bioactivities on cancer development, metastasis dissemination and angiogenesis. These preliminary results pave the way for further investigations in order to identify molecular and cellular targets of marennine and reinforce previous studies suggesting that marennine has promising bioactivities that could be beneficial for human health applications.

## Data Availability

Experimental data are available from the authors upon reasonable request.
